# The effects of eye movements on emotional memories: using an objective measure of cognitive load

**DOI:** 10.3402/ejpt.v7.30122

**Published:** 2016-07-04

**Authors:** Suzanne C. van Veen, Iris M. Engelhard, Marcel A. van den Hout

**Affiliations:** Department of Clinical Psychology, Faculty of Social and Behavioral Sciences, Utrecht University, Utrecht, The Netherlands

**Keywords:** EMDR, working memory, cognitive load, autobiographical memory, dual task, reaction times

## Abstract

**Background:**

Eye movement desensitization and reprocessing (EMDR) is an effective treatment for posttraumatic stress disorder. The working memory (WM) theory explains its efficacy: recall of an aversive memory and making eye movements (EM) both produce cognitive load, and competition for the limited WM resources reduces the memory's vividness and emotionality. The present study tested several predictions from WM theory.

**Objective:**

We hypothesized that 1) recall of an aversive autobiographical memory loads WM compared to no recall, and 2) recall with EM reduces the vividness, emotionality, and cognitive load of recalling the memory more than only recall or only cognitive effort (i.e., recall of an irrelevant memory with EM).

**Method:**

Undergraduates (*N=*108) were randomly assigned to one of three conditions: 1) recall relevant memory with EM, 2) recall relevant memory without EM, and 3) recall irrelevant memory with EM. We used a random interval repetition task to measure the cognitive load of recalling the memory. Participants responded to randomly administered beeps, with or without recalling the memory. The degree to which participants slow down during recall provides an index of cognitive load. We measured the cognitive load and self-reported vividness and emotionality before, halfway through (8×24 s), and after (16×24 s) the intervention.

**Results:**

Reaction times slowed down during memory recall compared to no recall. The recall relevant with EM condition showed a larger decrease in self-reported vividness and emotionality than the control conditions. The cognitive load of recalling the memory also decreased in this condition but not consistently more than in the control conditions.

**Conclusions:**

Recall of an aversive memory loads WM, but drops in vividness and emotionality do not immediately reduce the cognitive load of recalling the memory. More research is needed to find objective measures that could capture changes in the quality of the memory.

**Highlights of the article:**

- Recall of an aversive autobiographical memory is a cognitive demanding task.
- The vividness and emotionality of an aversive memory decrease more after recall with eye movements than after only recall or only cognitive effort (i.e., recall of an irrelevant memory with eye movements).
- The cognitive load of recalling the memory does not immediately reduce after recall with eye movements compared to only recall or only cognitive effort.
- Intervention duration is positively related to memory effects.

Eye movement desensitization and reprocessing (EMDR) is an effective treatment for posttraumatic stress disorder (PTSD; Chen et al., 2014; NICE, 2005). A central component of EMDR that distinguishes it from other trauma treatments is a dual focus of attention: the therapist asks the patient to recall a distressing memory while simultaneously making horizontal eye movements (EM; Shapiro, 2001). The scientific community responded with skepticism to the introduction of EMDR, which was not unjustified. For example, there was no clear theoretical rationale of how EM might alter traumatic memories and thereby contribute to EMDR's efficacy (Engelhard, 2012). Some scientists have argued that EMDR may simply be a variant of exposure therapy, in which the EM component has no additional effect (e.g., Herbert et al., 2000). Studies have used a laboratory paradigm to test whether EM has effects, in which participants recall an aversive autobiographical memory, with or without making EM. Before and immediately after the intervention, they recall the memory for a brief duration and then rate its vividness and emotional intensity (see Van den Hout & Engelhard, 2012). A recent meta-analysis has shown that memory vividness and emotionality decrease more after “recall with EM” than after “recall without EM” in healthy participants, and that EM have an additive effect in EMDR treatment studies (Lee & Cuijpers, 2013).

The effects of recall with EM cannot be explained by exposure alone. It can be explained by working memory (WM) theory (e.g., Andrade, Kavanagh, & Baddeley, 1997; Gunter & Bodner, 2008; Van den Hout & Engelhard, 2012). WM is a limited-capacity system that temporary maintains information in the service of current cognitive processing, such as reading, counting, or thinking (Baddeley, 2007). WM theory states that retrieving an aversive memory requires limited-capacity WM resources. If a dual task is performed (such as making EM) that also loads WM, fewer resources will be available for recall of the memory, rendering it less vivid and emotional. Through the process of reconsolidation, the blurred representation of the memory may be later recalled (Van den Hout & Engelhard, 2012).

Gunter and Bodner (2008) argued that if memory recall is indeed impaired by competition for the same WM resources, then other dual tasks that load WM should produce the same effects. This hypothesis has been confirmed by studies showing that, for instance, complex spatial tapping (Andrade et al., 1997), making vertical EM (Gunter & Bodner, 2008), performing serial subtractions (Engelhard, Van den Hout, & Smeets, 2011; Van den Hout et al., 2010), copying a complex drawing (Gunter & Bodner, 2008), attentional breathing (Van den Hout, Engelhard, Beetsma, et al., 2011), playing the computer game Tetris (Engelhard, Van Uijen, & Van den Hout, 2010), and attending to film clips (Tadmor, McNally, & Engelhard, in press), all produce similar blurring effects. The presumed common factor of these dual tasks is that they load WM. To test whether they do, we have proposed using reaction time (RT) tasks, in particular the random interval repetition (RIR) task (Vandierendonck, De Vooght, & Van der Goten, 1998; see Van den Hout & Engelhard, 2012). In a RIR task, participants respond to a randomly administered stimulus (e.g., a tone), with or without performing a dual task (Vandierendonck et al., 1998). The degree to which RTs slow down because of the dual task, compared to a single task condition (i.e., RIR only), provides a quantitative measure of the degree of cognitive load by that dual task.

Studies have shown that dual tasks that produced memory effects (i.e., EM, Tetris, mental arithmetic, attentional breathing, and film clips) also induced a substantial slowing down of RTs on RIR tasks (Engelhard et al., 2010; Engelhard, Van den Hout, & Smeets, 2011; Tadmor et al., in press; Van den Hout, Engelhard, Beetsma, et al., 2011; Van den Hout, Engelhard, Rijkeboer, et al., 2011), which implies that these tasks indeed load WM. Moreover, higher cognitive load (fast EM), as evidenced by increased RTs during a RIR task, resulted in larger memory effects than lower cognitive load (slow EM; Van Veen et al., 2015). In addition, passively listening to a series of tones does not substantially slow down RTs during a RIR task and is less effective (Van den Hout, Engelhard, Rijkeboer, et al., 2011; Van den Hout et al., 2012). In sum, these results are in line with the WM theory: dual task manipulation derives its effects from the loading of WM during recall of an aversive memory.

If the RIR task can measure subtle differences in cognitive load during execution of the dual task (Van Veen et al., 2015), then it may as well be able to measure differences in cognitive load during memory recall. The degree of cognitive load during memory recall may depend on memory vividness and emotionality. Scientists have presumed that cognitive load is higher for very vivid representations than for blurred representations (Baddeley & Andrade, 2000). Likewise, emotional material may grab more attention than neutral material, resulting in a higher cognitive load (Schimmack, 2005; Van Dillen & Koole, 2007). Van den Hout, Eidhof, Verboom, Littel, and Engelhard (2014, discussion) tested whether recall of an emotional memory requires more WM resources than recall of a neutral memory. Participants performed the RIR task under three conditions in a balanced order: RIR+recall of emotional memory, RIR+recall of neutral memory, and RIR task only. Recall of an emotional memory indeed produced the largest RTs, followed by recall of a neutral memory, and relative to RIR task only. Emotional and neutral memories both require WM resources, but emotional memories do this to a greater degree. To conclude, the RIR task provides an opportunity to study the cognitive load of recalling an aversive memory. Most EMDR analogue studies have used self-reported vividness and emotionality of the memory as dependent variables (see Lee & Cuijpers, 2013; Van den Hout & Engelhard, 2012). However, these measures may be susceptible to demand characteristics. Therefore, it has been argued that objective measures, like physiological measures or RT tasks, are needed (Engelhard et al., 2010; Kearns & Engelhard, 2015; Van den Hout, Bartelski, & Engelhard, 2013). The RIR task may potentially serve as such an objective measure.

The present study tested: 1) whether recall of an aversive memory loads WM compared to no recall, and 2) whether recall of an aversive memory while making EM (experimental condition) leads to decreases in cognitive load, vividness, and emotionality, compared to only recall (control condition 1) or only cognitive effort (control condition 2). To prevent unintended memory recall and resemble the cognitive effort from the experimental condition, participants in the second control condition recalled *another* (“irrelevant”) aversive memory while making EM. By including this condition, we also controlled for effects of training or fatigue on the RIR task. Participants were randomly assigned to one of three conditions in a between-subjects design: 1) “recall relevant memory with EM,” 2) “recall relevant memory without EM,” and 3) “recall irrelevant memory with EM.” To keep procedures equal, all participants selected two aversive memories, but only the third condition used both memories in the memory experiment. Note that cognitive load of recalling the memory was the measure of primary interest, but we also measured self-reported vividness and emotionality to establish comparability with earlier studies.

Although most studies have found that vividness and emotionality decrease after recall with EM compared to recall without EM (Lee & Cuijpers, 2013), some studies have failed to find an effect on *both* ratings (e.g., Engelhard, Van den Hout, & Smeets, 2011; Maxfield, Melnyk, & Hayman, 2008, exp. 1; Van den Hout, Engelhard, Beetsma, et al., 2011, exp. 2). These studies used a small number of intervention blocks (4–6×24 s; e.g., Engelhard, Van den Hout, & Smeets, 2011; Van den Hout, Engelhard, Beetsma, et al., 2011) or short intervention duration per block (10×8 s; Maxfield et al., 2008). Leer, Engelhard, and Van den Hout (2014) found drops in emotionality after eight blocks, but not after four blocks of intervention. In order to produce a subsequent reduction of vividness, emotionality, and cognitive load, we prolonged the intervention to 16×24 s. To get insight in the development over time, we measured the change in our dependent variables after the first intervention period (T1–T2; 8×24 s) and after the second intervention period (T2–T3; 8×24 s).

We used Bayesian model selection to measure the support for several prespecified models, derived from WM theory. We predicted that average RT during RIR+recall of the relevant memory (T1) would be higher relative to average RT during RIR task only (baseline) in all conditions (model 1). In the experimental condition, we expected that average RT during RIR+recall of the relevant memory and average visual analogue scale (VAS) scores of vividness and emotionality would decrease over time (model 2). Moreover, we expected these decreases to be larger than in the control conditions, from T1 to T2 (model 3) and from T2 to T3 (model 4). Finally, the full theoretical model was captured in model 5, which combines models 2–4. The hypothesis constraints can be found in the Appendix 1.

## Method

### Participants

Participants were undergraduate students from Utrecht University and the University of Applied Sciences (Hogeschool Utrecht), who received course credit or financial compensation for their participation. Prior to the experiment, individuals were excluded if they had visual or auditory problems, had participated in previous EMDR studies, had knowledge of how EMDR works, or used medication that affected memory or concentration. Seventeen individuals were excluded based on these criteria. Six participants were replaced: two could not select two vivid aversive memories and quit the experiment, and four experienced technical problems during the experiment, what may have influenced the data. The final sample consisted of 108 individuals (*M*_*age*_ *=*20.93, *SD_age_ =*2.17; 38 males, 70 females).

### Design

Participants underwent a baseline RIR task and were then randomized to one of three conditions: relevant with EM, relevant without EM, and irrelevant with EM. All participants selected two aversive autobiographical memories and selected a target image for each memory. Using counterbalancing, one memory was identified as “relevant memory” and the other memory was identified as “irrelevant memory.” For all participants, dependent variables were average RT on the RIR task during recall of the relevant memory and self-reported vividness and emotionality. Intervention (relevant with EM, relevant without EM, irrelevant with EM) was a between-subjects factor and time (T1–T3) was a within-subjects factor.

### Materials and procedure

**Fig. 1:**
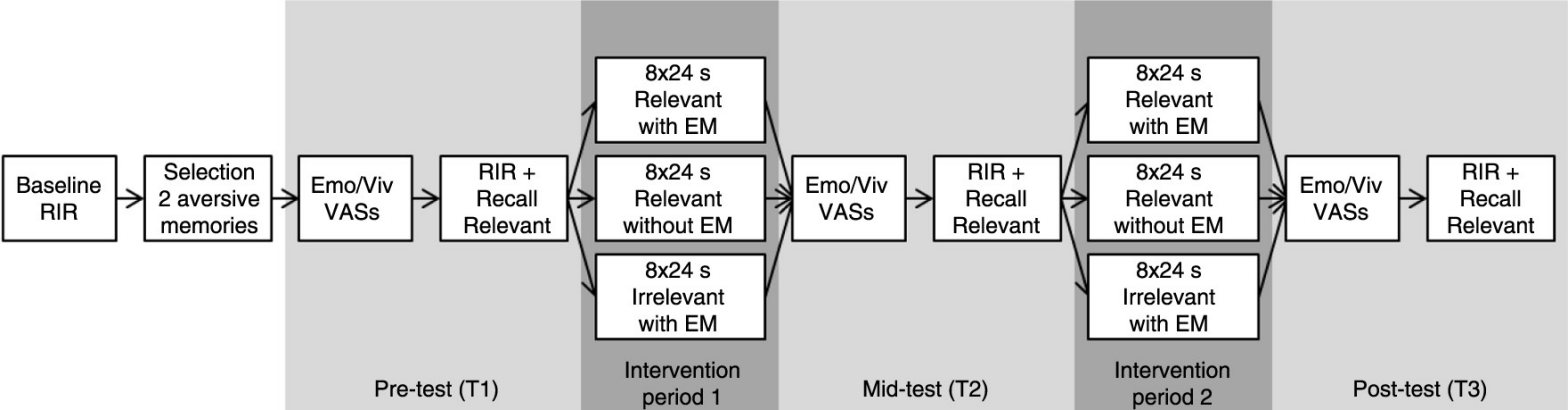
Timeline of experimental procedure. In the relevant memory conditions (with or without EM), the *irrelevant* memory was generated at baseline to ensure comparability with the irrelevant memory with EM condition. In the relevant memory conditions, the irrelevant memory was not activated during the experiment proper.

After screening and informed consent, participants were seated behind a computer with a screen resolution of 1,280×1,024 at a distance of about 45 cm, and wore headphones during the tasks. OpenSesame 2.8.3 (Mathôt, Schreij, & Theeuwes, 2012) was used to present instructions and stimuli. Prior to the main experiment, participants completed a practice session to familiarize them with the RIR task (20 beeps; 24 s), the EM task (24 s), and the black screen task (24 s). Figure 1 shows a timeline of the experimental procedures.

In the RIR task (Vandierendonck et al., 1998; cf. Van den Hout, Engelhard, Beetsma, et al., 2011), participants responded as quickly as possible to beeps (200 Hz) by pressing the *b*-key on the keyboard. Beeps were administered in both ears at a clearly detectible, constant volume. Each beep was presented for 50 ms, and the inter-stimulus interval was quasi-random 900 or 1,500 ms measured from the onset of the beep to the onset of the following one. The intervals were sampled randomly with a probability of 0.50, and the restriction of no more than four of the same intervals in a row. Dependent variable was average RT, and we also observed error rates (failure to respond in time to the beep). For calculation of the average RT, the responses to the first and last beeps were always left out, to exclude potential transition delays. We did not remove any outliers.

The EM task consisted of a 1 cm white dot moving horizontally across a black screen at a rate of 1 left–right–left cycle per second (1 Hz). Movement amplitude was 30 cm. Participants held their head still while following the white dot with their eyes.

During the black screen task, participants held their head still while looking at a black screen. To minimalize WM taxation, no fixation cross was used.

After the practice sessions, participants conducted the baseline RIR task, which consisted of 40 beeps (28 s). Participants were then seated at a table for the memory and target image selection. Participants selected two vivid, negative memories of at least 1 week old that still evoked feelings of distress when they were recalled in the here and now (cf. Van den Hout, Muris, Salemink, & Kindt, 2001). Participants recalled, for example, the illness or death of a family member, a traffic accident, or break-up with a loved one. They wrote down the content of each memory on a card and rated the vividness and emotionality of each memory on two 0–100 scales (0=*not at all vivid/unpleasant*, 100=*very vivid/unpleasant*). Vividness was defined as “memories that are very clear and detailed,” and emotionality was defined as “memories that give you an unpleasant feeling when you recall them.” Selection criteria were a score of 70 or above for vividness and 50–90 for emotionality. For ethical reasons, we used a score of 90 as upper limit for emotionality. We based the other selection criteria on the results of Van den Hout et al. (2013). Memories were ranked based on vividness ratings. The selection of the *relevant* memory was counterbalanced based on the vividness rankings.

The target image selection was almost identical to the Dutch EMDR standard protocol (De Jongh & Ten Broeke, 2012). For all participants, target image selection took place for both memories (i.e., relevant and irrelevant), and the order of the selection was counterbalanced based on the vividness ratings. Participants described the memory in global story lines, identified the worst moment of the memory (“hotspot”) as a still image, and assigned a relatively neutral label to this specific image (for example, “red car” or “statistics exam”). All instructions of the memory experiment (VAS ratings, RIR task, EM task, black screen task) were digitalized and included the label of the target image, to ensure that participants knew which memory image they had to recall.

For the memory experiment, participants sat behind the computer again and wore headphones. The experimenter took place besides the participant to observe the movements of the eyes of the participant during the EM task or black screen task. At pretest (T1), participants first recalled the image of the relevant memory for 10 s and rated its vividness and emotionality on two VASs that ranged from 0 (*not at all vivid/upleasant*) to 100 (*very vivid/unpleasant*). Next, they recalled the image of the relevant memory for 48 s while performing the RIR task (40 beeps). During the first intervention period, participants received eight blocks of 24 s intervention with 10 s breaks in between (262 s in total). In the relevant with EM condition, participants recalled the image of the *relevant* memory while performing the EM task. In the relevant without EM condition, participants recalled the image of the *relevant* memory while performing the black screen task. In the irrelevant with EM condition, participants recalled the image of the *irrelevant* memory while performing the EM task. After the first intervention period, the midtest (T2), second intervention period, and posttest (T3) took place, which resembled earlier procedures. Finally, participants were debriefed and given their reward.

### Data analysis

The hypotheses were evaluated using Bayesian model selection. Benefits of Bayesian statistics are that complex models can be tested at once, and it does not depend on dichotomous decisions (i.e., the result is significant or not) but rather defines the relative support for a prespecified model (Van de Schoot & Depaoli, 2014). The results of the Bayesian model selection are expressed in terms of Bayes factor (BF). In our study, BF represents the amount of evidence provided by the data in favor of the model (“H1”) compared to its compliment (“not H1”; Van Rossum, Van de Schoot, & Hoijtink, 2013). A BF of 1 means that, compared to its compliment, the model has equal support. A BF<1 indicates that there is no support for the model by the data, whereas a BF>1 means that the model is supported by the data. The higher the BF, the more the model is supported by the obtained data. Furthermore, BF>10 reflects “strong” evidence for the model (see Jeffreys, 1961, for a classification scheme for the BF). Analyses were performed using the software BIEMS (see Mulder, Hoijtink, & De Leeuw, 2012). For further reading about Bayesian analysis and the comparison with *p*-value significance testing, we recommend Wetzels et al. (2011) and Krypotos, Blanken, Arnaudova, Matzke, and Beckers (2016).

Table 1 provides descriptive statistics for RTs, emotionality and vividness. We observed the average number of errors during the RIR task, and these were low at all time points for all conditions.

**Table 1:** Means and standard deviations for RTs, vividness and emotionality before (T1), halfway through (T2), and after (T3) recall relevant memory with EM (“relevant with EM”), recall relevant memory without EM (“relevant without EM”), and recall irrelevant memory with EM (“irrelevant with EM”)

	RTs		
	
	T1	T2	T3

Relevant with EM	413.86 (*140.50*)	384.11 (*128.99*)	360.71 (*109.90*)
Relevant without EM	373.13 (*113.31*)	322.47 (*68.80*)	345.99 (*91.94*)
Irrelevant with EM	344.32 (*73.14*)	327.22 (*62.30*)	329.10 (*64.15*)

	Vividness		
	
	T1	T2	T3

Relevant with EM	75.36 (*20.63*)	63.66 (*20.60*)	55.08 (*23.02*)
Relevant without EM	70.53 (*22.42*)	67.64 (*21.30*)	62.94 (*24.73*)
Irrelevant with EM	76.52 (*15.84*)	66.82 (*18.93*)	63.22 (*21.75*)

	Emotionality		
	
	T1	T2	T3

Relevant with EM	67.43 (*20.72*)	60.79 (*20.78*)	48.48 (*23.05*)
Relevant without EM	68.95 (*17.99*)	63.72 (*22.27*)	56.55 (*23.34*)
Irrelevant with EM	69.29 (*20.21*)	66.57 (*21.52*)	59.87 (*21.00*)

*Note*. We did not correct for outliers; EM=eye movements, RT=reaction time.

## Results

### Cognitive load of recalling the memory

**Fig. 2:**
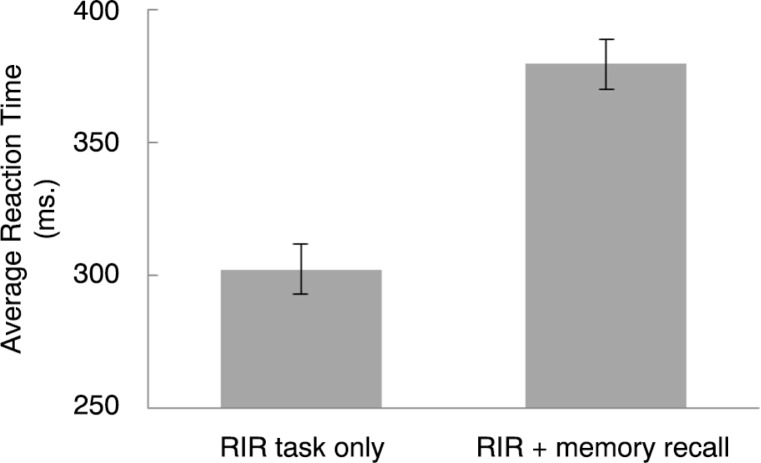
Average RTs (ms.) and SEMs (adjusted for within-group comparison) for RIR task only (baseline) and RIR + memory recall (T1).

Figure 2 represents the average RT for RIR task only (baseline) and RIR+memory recall (T1). The figure shows that, in line with model 1, RTs increased when participants recalled the relevant memory during the RIR task, compared to RIR task only. This was also strongly supported by the BF value (BF=952.27).

### Reductions in cognitive load of recalling the memory over time

**Fig. 3:**
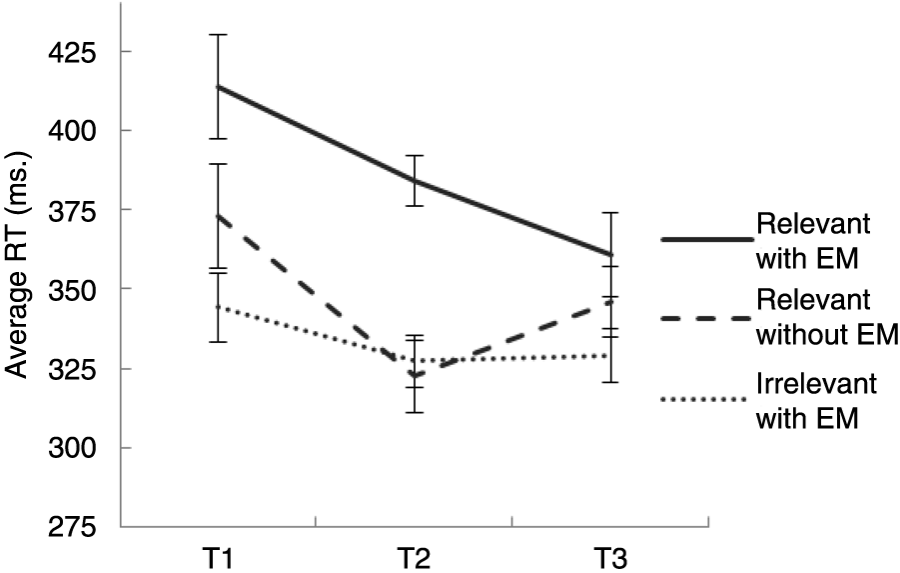
Average RTs (ms.) and SEMs (adjusted for within-group comparison) before (T1), halfway through (T2), and after (T3) recall relevant memory with EM (“relevant with EM”), recall relevant memory without EM (“relevant without EM”), and recall irrelevant memory with EM (“irrelevant with EM”).

Figure 3 shows the average RT on the RIR task over time per condition. Unexpectedly, we observed pretest (T1) differences between conditions on the RIR task. To correct for these differences, we analyzed models 2–5 with the centered average RT at T1 as covariate. The decrease of RT over time in the recall relevant memory with EM condition was strongly supported by our data (model 2; BF=16.81). However, RT reductions in the recall relevant memory with EM condition were only larger than in the control conditions after the second intervention period (model 4; 10.49) but not after the first intervention period (model 3; BF=0.05). Therefore, the full theoretical model was not supported by the data (model 5; BF=0.49).

### Reductions in vividness and emotionality over time

**Fig. 4:**
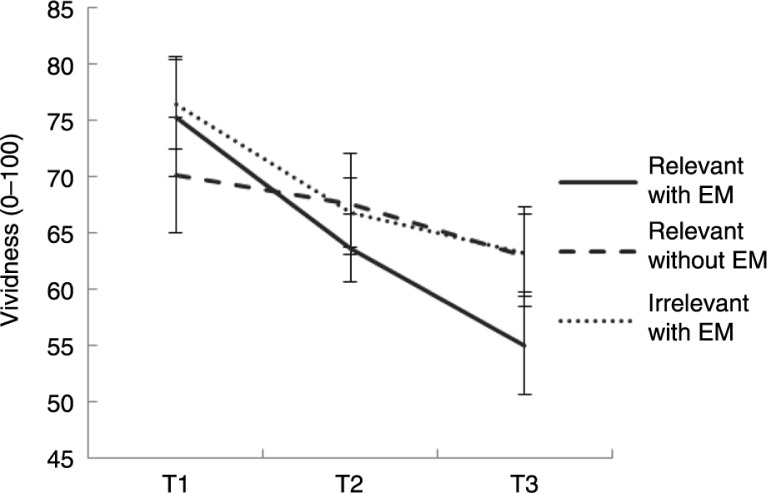
Average vividness and SEMs (adjusted for within-group comparison) before (T1), halfway through (T2), and after (T3) recall relevant memory with EM (“relevant with EM”), recall relevant memory without EM (“relevant without EM”), and recall irrelevant memory with EM (“irrelevant with EM”).

**Fig. 5:**
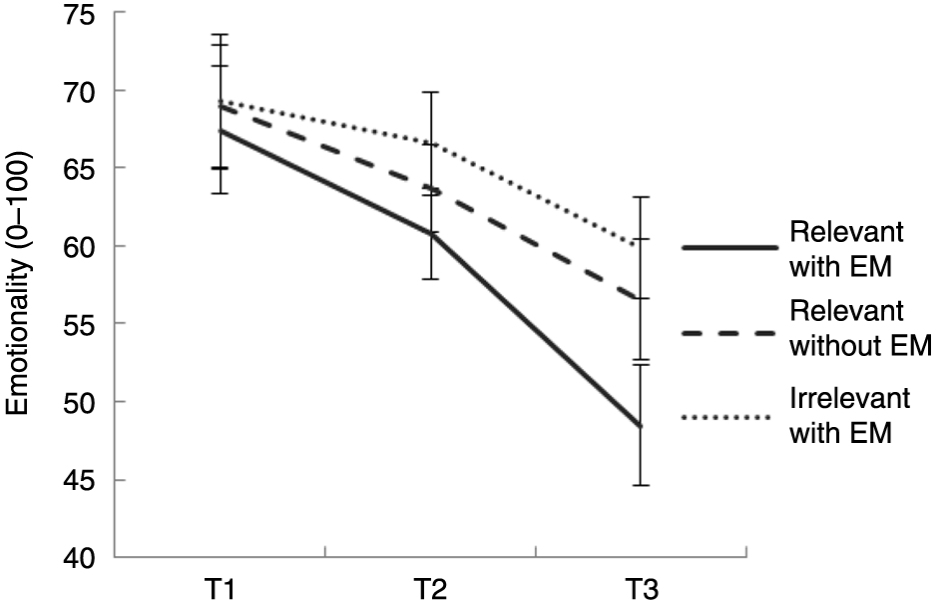
Average emotionality and SEMs (adjusted for within-group comparison) before (T1), halfway through (T2), and after (T3) recall relevant memory with EM (“relevant with EM”), recall relevant memory without EM (“relevant without EM”), and recall irrelevant memory with EM (“irrelevant with EM”).

Models 2–5 were also analyzed for vividness and emotionality data. Figures 4 and 5 represent the average scores on the vividness and emotionality VASs over time per condition. We found strong evidence that when the relevant memory was recalled while making EM, vividness (BF=485.12) and emotionality (BF=145.09) decreased over time. After the first intervention period (T1–T2), we found a mild preference for model 3 both for vividness (BF=2.85) and emotionality (BF=2.25) ratings; that is, these ratings decreased more in the recall relevant memory with EM condition than in the control conditions. After the second intervention period (T2–T3), the support for the superiority of the recall relevant with EM condition was stronger: BF=5.09 for vividness and BF=10.36 for emotionality. The full theoretical model was strongly supported by the data for vividness (BF=18.21) and emotionality (BF=19.28).

## Discussion

This study tested several predictions from the WM theory, using objective and subjective measurements. Our findings provide insight into four issues related to the dual task intervention: the magnitude of cognitive load of recalling the memory, the effect of dual task intervention on this cognitive load, the role of intervention duration in dual task intervention, and the importance of active recall during task processing. We will address these issues below.

### The magnitude of cognitive load of recalling the memory

A basic assumption of WM theory is that recall of a memory is a cognitive demanding process that loads limited WM recourses. Testing the feasibility of this assumption can be hard when one uses subjective measures. A RT task, like the RIR task (Vandierendonck et al., 1998), is a simple and sensitive method that has been used in previous studies to measure differences in cognitive load. For example, making EM is more demanding than passively listening to beeps (Van den Hout, Engelhard, Rijkeboer, et al., 2011), playing Tetris is more demanding than making EM (Engelhard et al., 2010), and making fast EM is more demanding than making slow EM (Van Veen et al., 2015). In line with the preliminary findings of Van den Hout et al. (2014), we found that RTs were slower when the RIR task was performed during memory recall compared to RIR only. Relative to RIR only, cognitive load of recalling the memory (*M*_*dif*_=77 ms) is comparable with cognitive load of making EM (*M*_*dif*_=87 ms; Van den Hout, Engelhard, Beetsma, et al., 2011). In sum, we conclude that recall of an autobiographical aversive memory indeed loads WM resources.

### The effect of dual task intervention on cognitive load of recalling the memory

We predicted that memories rendered less vivid and emotional by dual task intervention would impose a smaller cognitive load than memories in control conditions. This prediction was only partially supported by our data. In accordance with the reductions on the subjective measures, we found a reduction of cognitive load when the memory was repeatedly recalled while making EM. This reduction was larger in the experimental condition than in the control conditions after the second intervention period, but not after the first intervention period. These findings mirror the reductions in vividness and emotionality to some extent, as these changes were not as pronounced after the first intervention period as they were after the second intervention period. Important questions are why we did not find the predicted effects after the first intervention period and how we should interpret the similarities and differences in effects on the objective and subjective measures. One conclusion might be that even though recall of a memory and making EM both load WM resources, this dual tasking might not necessarily decrease the cognitive load of recalling the memory. However, studies have shown that emotional memories require more WM resources than neutral memories (Van den Hout et al., 2014), and that vividness is positively related to cognitive load (Baddeley & Andrade, 2000). On the other hand, Van den Hout et al. measured differences between emotional and *neutral* memories, whereas we measured differences between very emotional/vivid memories and moderately emotional/vivid memories. One could argue that it may have been overly restrictive to predict differences between conditions in cognitive load after the first intervention period. Therefore, replication studies that measure the cognitive load of recalling the memory before and after substantial reductions (e.g.,<20 on scale 0–100) in vividness and emotionality are warranted.

### The role of intervention duration in dual task intervention

Our study confirms previous findings that performing a cognitive demanding task during recall of an aversive memory reduces the vividness and emotionality of that memory during future recall (see Van den Hout & Engelhard, 2012). In line with Leer et al. (2014), our results provide evidence that intervention duration is positively related to the memory effects. Instead of manipulating the intervention duration between conditions, we prolonged the invention duration and measured vividness and emotionality of the memory over time. Our results suggest that the memory effects are more evident after the second than after the first intervention period. If competing cognitive load causes vividness/emotionality reductions because vividness/emotionality consume WM resources, why would this not be observed after the first intervention period? That is, why would a longer intervention be more beneficial? The Time-Based Resource-Sharing (TBRS) model (Barrouillet, Bernardin, & Camos, 2004) may explain this finding. Similar to WM theory, a first assumption of the TBRS model is that maintenance and processing of information rely on the same limited WM resources and require the same controlled attention. Second, the TBRS model posits that only one cognitive process can occur at a time. Third, activated information (e.g., a memory) suffers from a time-related decay when attention is switched away (Barrouillet et al., 2004). When individuals do not refresh their memory recall, the memory slowly fades away. Only through rapid and repeated switching between both tasks, a memory can remain activated. This is a demanding process, so any increase in the duration of the intervention should lead to an increase in cognitive load and hence poorer recall of the memory. To conclude, cognitive load seems to depend not only on the complexity of the dual task (Van Veen et al., 2015), but also on the duration of the dual task procedure. Future studies could test the TBRS model by comparing two conditions that are similar in intervention duration but differ in the ease of memory activation (e.g., no breaks in between vs. breaks in between activation phases). We further recommend extending the intervention duration of the EMDR analogue paradigm (e.g., from 4×24 s to 16×24 s).

### The importance of active recall during task processing

Previous EMDR analogue studies used a recall only condition as the control condition (see Van den Hout & Engelhard, 2012). Comparison between recall with or without making EM provides insight in the additional value of making EM. The superiority effect of recall with EM could be explained by the WM theory, but comparison between these two conditions does not rule out the possibility that cognitive effort itself accounts for the larger memory effects. In addition to a recall only condition, we included a control condition in which participants recalled another “irrelevant” aversive memory while making EM. The recall “relevant” with EM condition produced larger reductions in vividness and emotionality than both control conditions. This finding supports the idea that the memory effects are caused by the dual task procedure and not by mere recall or general cognitive effort. This finding is also important, because in some earlier studies that compared the effects of recall with a dual task to recall only, the differential effects of the conditions were (partly) driven by increases in emotionality and vividness in the recall only condition, rather than decreases in the recall with dual task condition (e.g., Engelhard, Van den Hout, Dek, et al., 2011).

WM theory states that memory recall and making EM should be performed simultaneously for competition between these tasks to occur. However, some recent studies showed that recall of emotional memories *followed by* a complex task (e.g., reading a story, Kredlow & Otto, 2015; or playing the computer game Tetris, James et al., 2015) decreased the number of details or intrusiveness of the memory compared to only recall, only inference, or none. The authors explained these effects by the process of retroactive interference. It is known that recalled memories could enter a labile phase, in which the memory can be disrupted before a new process of stabilization called reconsolidation takes place (Agren, 2014). During this labile phase, execution of a complex task may retroactively interfere with the memory. Observation of the data in our study shows a beneficial effect of memory recall *during* interference compared to interference *after* memory recollection. Nevertheless, vividness and emotionality also seemed to decrease somewhat in the recall irrelevant with EM condition. This could still be the result of retroactive interference. Future studies may further compare the effects of interference during or after memory recollection, and usea posttest after the reconsolidation window is closed.

### Study limitations

This study has some limitations. First, we observed unexpected pretest (T1) differences between conditions on the RIR task. Therefore, we analyzed the RT models with the centered RT at T1 as covariate. Because we randomized participants to conditions and used counterbalancing procedures within each condition, it remains unclear how these pretest differences may have occurred. Second, the selection of the relevant memory and the order of the target image selection were counterbalanced based on vividness scores. Considering the range of scores used as selection criteria for vividness (> 70) and emotionality (50–90), it would have been better to rank memories based on emotionality scores and use this ranking for counterbalancing. Third, prior to the main experiment, participants received a short practice session including the EM and black screen task. Although participants had no prior knowledge of EMDR and were not informed about the number and content of the conditions, we cannot rule out that this practice session influenced the participants’ expectations. In future studies, we suggest using a tailored practice session per condition. Fourth, we did not measure the resemblance in content between the relevant and irrelevant memories, and did not ask whether recall of the irrelevant memory in the third condition also triggered the relevant memory. The observed small reductions in this condition may have resulted from unintended activation of the relevant memory during the intervention periods. In future studies that include two memories, researchers could ask participants to select two memories that differ in content and retrospectively measure the unintended activation of both memories.

## Conclusion

In accordance with the WM theory, we found strong support that recall of aversive memories loads WM resources, and that simultaneous execution of memory recall and making EM leads to memories that are less vivid and emotional, compared to only recall and only cognitive effort (i.e., recall with EM on another memory). Crucially, cognitive load of recalling the memory decreased after recall with EM but not consistently more than after only recall and only cognitive effort. More research is needed to investigate whether immediate decreases in vividness and emotionality reflect permanent changes in the quality of the memory, and to search for measures that can capture such changes.

## Appendix 1

Hypothesis constraints for RTs (models 1–5), vividness (models 2–5) and emotionality (models 2–5)

**Table:**

Model	Constraints
Model 1	RTs increase during memory recall compared to no recall.
	µ RT (T1) > µ RT (Baseline)
Model 2	Reductions in relevant with EM condition over time.
	µ T1 > µ T2 > µ T3
Model 3	T1 to T2 reduction is larger in relevant with EM condition than in control conditions.
	$\left(\begin{array}{c} \text{Relevant with EM}\\ \mu \text{T1}-\mu \text{T2} \end{array}\right)>\left(\begin{array}{c} \text{Relevant without EM}\\ \mu \text{T1}-\mu \text{T2} \end{array}\right)$ $\left(\begin{array}{c} \text{Relevant with EM}\\ \mu \text{T1}-\mu \text{T2} \end{array}\right)>\left(\begin{array}{c} \text{Relevant without EM}\\ \mu \text{T1}-\mu \text{T2} \end{array}\right)$
	
Model 4	T2 to T3 reduction is larger in relevant with EM condition than in control conditions.
	$\left(\begin{array}{c} \text{Relevant with EM}\\ \mu \text{T1}-\mu \text{T2} \end{array}\right)>\left(\begin{array}{c} \text{Relevant without EM}\\ \mu \text{T1}-\mu \text{T2} \end{array}\right)$ $\left(\begin{array}{c} \text{Relevant with EM}\\ \mu \text{T1}-\mu \text{T2} \end{array}\right)>\left(\begin{array}{c} \text{Relevant without EM}\\ \mu \text{T1}-\mu \text{T2} \end{array}\right)$
	
Model 5	Models 2–4 combined.
